# Influence of Protein Intake during Complementary Feeding on Body Size and IGF-I Levels in Twelve-month-old Infants

**DOI:** 10.4274/balkanmedj.galenos.2019.2019.6.5

**Published:** 2019-12-20

**Authors:** Iulia Florentina Ţincu, Daniela Păcurar, Radu Ciprian Ţincu, Cristina Becheanu

**Affiliations:** 1 “Carol Davila” University of Medicine and Pharmacy, Bucharest, Romania; 2 Department of Pediatrics, “Grigore Alexandrescu” Emergency Children’s Hospital, Bucharest, Romania; 3 Critical Care Toxicology Unit, Bucharest Clinical Emergency Hospital, Bucharest, Romania

## To the Editor,

Our understanding of postnatal growth and later influence on childhood obesity by early nutrition is furnished with a growing evidence worldwide. Early dietary risk factors should be considered to prevent later obesity (1). Few epidemiological trials have investigated the role of protein intake during the weaning period in stimulating the endocrine and metabolic mechanisms that further lead to rapid weight gain and adiposity in children. This paper examines whether protein intake from complementary feeding is associated with insulin-like growth factor-I (IGF-I) levels and greater growth rates in 12-month-old infants.

The eligible population for the study consisted of healthy term infants examined at their check-up visits at 6 and 12 months. Of the 127 families of infants invited to participate in the study, only 75 (85%) completed the 1 year follow-up period. All participants were assessed with anthropometry, 3 days food record, and blood analysis of IGF-I at the age of 12 months.

The distributions according to Z-score for weight-for-length in terms of underweight, normal weight, and overweight/obese children were 11.5%, 74.3%, and 14.2%, respectively, at the age of 12 months. Girls had considerably higher levels of IGF-I than boys [78.3 ng/mL (51.3 ng/mL; 114.2 ng/mL) vs 72.5 ng/mL (49.3 ng/mL; 92.1 ng/mL)]. There was a progressive trend of decreasing partial or full breastfeeding according to age, with 55% of infants being breastfed for 4 months.

The median protein intake was 3.4 g/kg of body weight, ranging between 1.1 and 5.2 g/kg body weight. Daily protein intake at the age of 12 months was positively correlated with body size in terms of weight and length (p<0.0001). Protein intake was significantly correlated with serum urea and IGF-I levels. We calculated the determination coefficient to analyze the effect of protein intake on IGF-I levels, meaning R^2^=70.5%.

The infants receiving <2.5 g protein/kg of body weight had median levels of IGF-I to be 35% lower than the ones receiving higher amounts. Serum concentrations of urea and glucose were also significantly higher in infants fed with higher amount of protein in their diet (Table 1). Also, infants being breastfed for 6 months had lower IGF-I levels at the age of 12 months than those receiving formula [76.6 ng/mL (52.8 ng/mL; 113.5 ng/mL) and 71.3 ng/mL (48.1 ng/mL; 89.3 ng/mL), p<0.05].

This is the first analysis of quantitative diet received by Romanian infants; although, descriptive data have been reported in a previous investigation (2). Rolland-Cachera et al. (2) were the first to introduce the early protein hypothesis stating that high protein intake in early infancy induces obesity risk later in life. The protein content of the diet offered to the infants was quite high compared to normal ranges (: 15% of energy; 90th percentile: 19% of energy); some other studies report data on protein intake in late infancy to be 16.3% (3) and 20% of energy (4). The larger the amount of daily protein intake that the infants were offered, the more their weight and height were at the age of 12 months.

The introduction of solid food in children’s diet offers increasing amount of protein, sometimes exceeding real needs. Further studies on extended population are needed to provide sustained evidence about the impact of protein amount during complementary growth rates in childhood.

## Figures and Tables

**Table 1:**
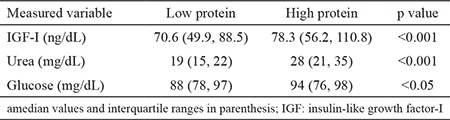
Variation of IGF-I, urea, and glucose levels according to protein intakea
